# Deep Eutectic Solvents Formed by Glycerol and Xylitol, Fructose and Sorbitol: Effect of the Different Sugars in Their Physicochemical Properties

**DOI:** 10.3390/molecules28166023

**Published:** 2023-08-11

**Authors:** Laura Lomba, Álvaro Werner, Beatriz Giner, Carlos Lafuente

**Affiliations:** 1 Facultad de Ciencias de la Salud, Campus Universitario, Universidad San Jorge, Autov. A23 km 299, Villanueva de Gállego, 50830 Zaragoza, Spain; llomba@usj.es (L.L.); werneralvaro@gmail.com (Á.W.); 2 Departamento de Química Física, Facultad de Ciencias, Universidad de Zaragoza, 50009 Zaragoza, Spain; celadi@unizar.es

**Keywords:** deep eutectic solvents, physicochemical properties, glycerol, sugars

## Abstract

The search for new eutectic solvents for different applications (extraction, drug formulation, chemical reactions, etc.) is booming thanks to their high solubility capacity and low toxicity. However, it is necessary to carry out a comprehensive physicochemical characterization of these mixtures to understand the molecular behavior at different experimental conditions. In this study, three deep eutectic solvents (DESs) formed by glycerol and xylitol, fructose and sorbitol and water in the molar ratio 1:2:3 were prepared and several physicochemical properties (refractive index, density, surface tension, viscosity, speed of sound, isobaric heat capacity and isentropic compressibility) were measured and analyzed in the 278.15–338.15 K temperature range. The results indicate a linear dependence with temperature for the following properties: surface tension, refractive index, density and isobaric molar heat capacity while viscosity values have been fitted to the Vogel–Fulcher–Tammann equation.

## 1. Introduction

It is well known that solvents play an important role in chemical processes. In addition to fulfilling their primary function (dissolving solid substances in a liquid medium), solvents are used to reduce the viscosity of the system and as reaction media. Furthermore, they can be used for important applications such as extraction or even in the pharmaceutical and food industries [1]. Traditional organic solvents have been used over the years due to their good chemical properties that facilitate the processes in which they are involved; however, there are several disadvantages associated with their use, such as flammability, toxicity, volatile character, or low biodegradability profiles [2]. As a result, there is a growing environmental concern that has led to the search for and development of safer and more sustainable alternatives.

One of these new options which is now at the peak of its popularity is deep eutectic solvents (DESs). First described by Abbott et al. [3], DESs are traditionally formed by a hydrogen acceptor substance (HBA) and a hydrogen donor substance (HBD) [4]. Typical substances acting as HBAs in DESs are betaine, menthol, thymol, choline chloride or lauric acid, among many others [5]. On the other hand, urea, amino acids (serine, glutamate, proline, alanine), amines, alcohols (1,2-propylene glycol, xylitol, glycerol, sorbitol or ethylene glycol), sugars (sucrose, fructose, glucose, lactose, xylose or galactose), amides (benzamide or acetamide) or carboxylic acids (tartaric, lactic, oxalic, malonic, maleic, citric or malic) can be used as HBDs [6,7]. All these raw materials can be of sustainable origin, and then we have the so-called natural deep eutectic solvents, NADESs. One of the most attractive features of these systems is the tunability of their physicochemical properties, which reflect the extent of the strong hydrogen bonding interactions between the molecules [8]. However, these systems are sometimes difficult to work with because NADESs generally have higher viscosity values than traditional solvents due to an increase in the hydrogen bonds formed between the components of the system. However, to reduce the viscosity of this type of system, it is usually possible to change the molar ratio and the temperature, or to add small amounts of water to make the system more manageable [9]. They are also non-flammable, have good chemical and thermal stability and have low volatility and vapor pressure. Finally, although toxicity depends on the HBAs and HBDs used, they are said to be non-toxic to both the environment and human health. All this, together with the possibility of solvent recovery after a certain application, has earned these solvents the “green” label, and NADESs are being widely used under the umbrella of the circular economy. In addition, the nature and versatility of NADESs allow them to be used in a wide range of applications, being better than DESs for co-extract elements from plant materials. This is one of the additional advantages of NADESs. For instance, they have been used to extract different classes of compounds such as phlorotannins [10], steroidal saponins such as borassoside E and protodioscin [11], triterpenoid saponins from *Aralia elata* var. *mandshurica* [12], polysaccharides [13], anthocyanins [14], phenyletanes and phenylpropanoids [15]. Furthermore, they can be used in organic synthesis [16,17,18], separation processes [19], biocatalysis [20,21], nanomaterials, biotechnology [6,22], electrochemistry [6,22,23], the food industry [24] or, in the case of the pharmaceutical industry, to increase drug solubility [25,26], used as drug delivery systems [27,28], for the treatment of tuberculosis [29] or for the treatment of rosacea [30].

In this study, a physicochemical characterization of three DESs formed by glycerol and mixed with fructose, xylitol or sorbitol and water in a molar ratio of 1:2:3 (sugar/glycerol/water) was carried out with the aim of analyzing the effect of the sugar on the behavior of the system. In this case, all the components of the eutectic system can act as HBAs and HBDs, as they all have hydrogen donor bonding sites. Several properties such as surface tension, refractive index, viscosity, density and isobaric molar heat capacity were obtained in the temperature range of 278.15–338.15 K and at *p* = 0.1 MPa. Several derived properties were calculated from experimental data.

The accurate determination of these physical and chemical properties is essential for the development and optimization of technologies, industrial processes and products in various scientific and engineering fields. These properties provide valuable information about the behavior of materials and substances, allowing informed decisions to be made for the advancement of science and technology. Knowing how these systems behave through different physicochemical properties makes it possible to know which systems to choose for each application [31], for example, if one system is better than another for extracting or separating certain compounds, or even if it has better properties for use in the biomedical field, such as enhancing drug solubility.

## 2. Results and Discussion

### 2.1. Preparation of DES

The studied deep eutectic solvents formed a transparent homogeneous solution, which remained as it was over time, with no precipitate appreciation at any of the studied temperatures.

### 2.2. Thermophysical Properties

**Figure 1:**
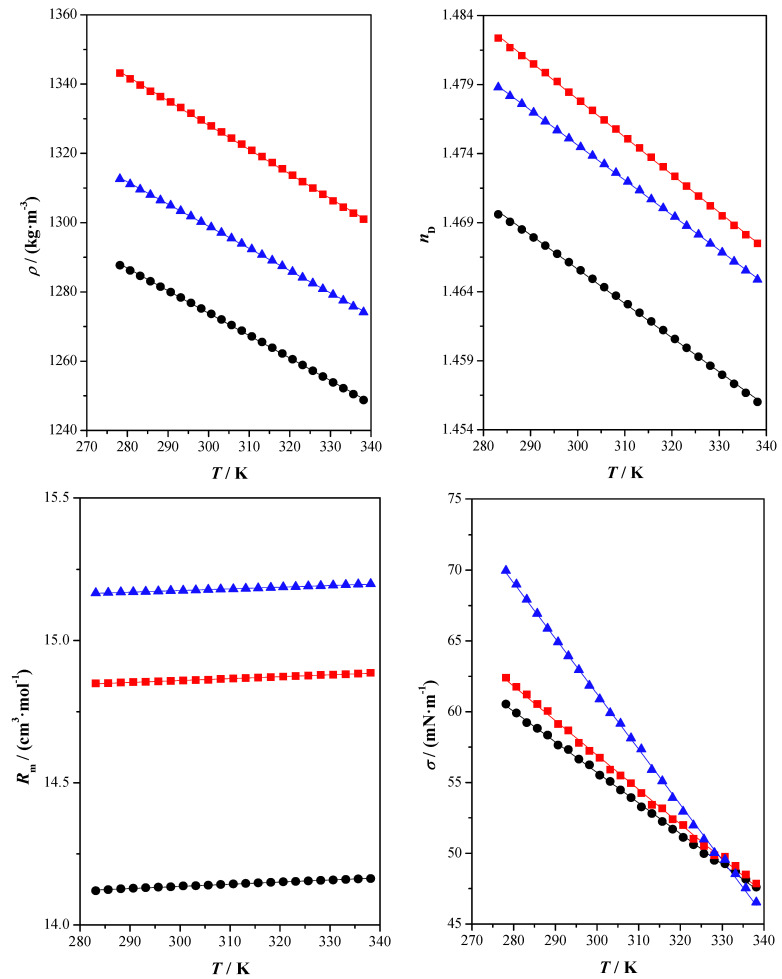
Density, *ρ*, refractive index, *n*_D_, molar refraction, *R_m_*, and surface tension, *σ*, as a function of temperature, *T*, at atmospheric pressure, *p* = 0.1 MPa for the studied compounds: xylitol: glycerol: water (●) experimental; fructose: glycerol: water (■) experimental; sorbitol: glycerol: water (▲) experimental; (^____^) correlated values.

**Figure 2:**
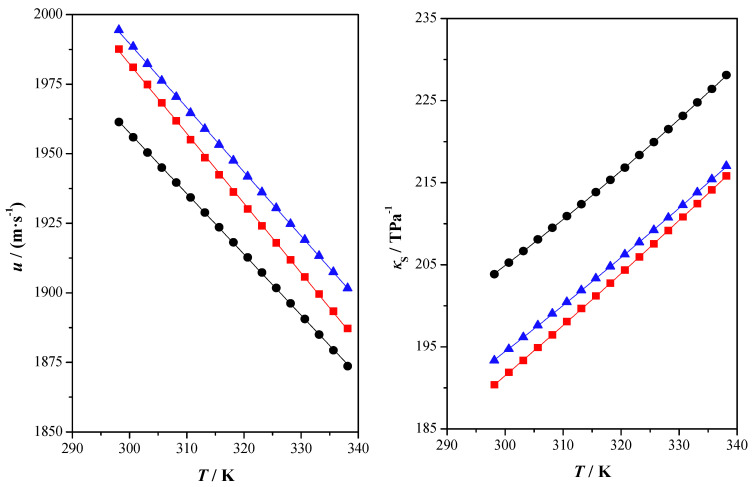
Speed of sound, *u*, and isentropic compressibility, *κ*_S_, as a function of temperature, *T*, at atmospheric pressure, *p* = 0.1 MPa for the studied compounds: xylitol: glycerol: water (●) experimental; fructose: glycerol: water (■) experimental; sorbitol: glycerol: water (▲) experimental; (^____^) correlated values.

**Figure 3:**
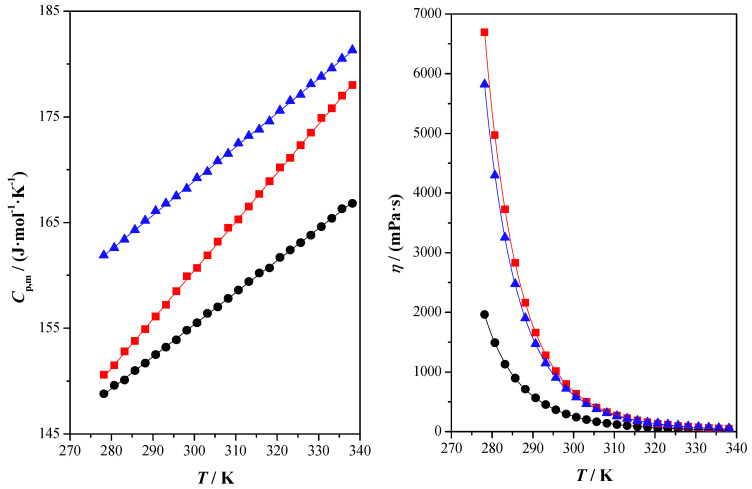
Isobaric molar heat capacity, *C*_p,m_, and dynamic viscosity, *η*, as a function of temperature, *T*, at atmospheric pressure, *p* = 0.1 for the studied compounds: xylitol: glycerol: water (●) experimental; fructose: glycerol: water (■) experimental; sorbitol: glycerol: water (▲) experimental; (^____^) correlated values.

Table S1 of the Supplementary Materials shows experimental measured and calculated data of the studied properties. This information is graphically shown in Figure 1, Figure 2 and Figure 3.

The relationship between the experimental data obtained for each property and the temperature was also obtained. A linear dependence with the temperature was observed for the following properties: refractive index, speed of sound, surface tension, density and isobaric molar heat capacity. The experimental data were correlated with the following linear equation:

(1) y=AT+B

where *Y* is the corresponding property and *A* and *B* are adjustable parameters.

The Vogel–Fulcher–Tammann equation was used to fit dynamic viscosity values [32,33,34]:

(2) $$\eta =\eta_{0}e^{\frac{B}{\left(T-T_{0}\right)}}$$

where *η_0_*, *B* and *T*_0_ are the fitting parameters.

Table 1 shows the fitting parameters for these fittings and the corresponding standard deviations, *S_yx_*, between the experimental and calculated values for each studied property. Standard deviations, *S_yx_*, were calculated using the next mathematical expression:

(3) $$S_{yx}={\left(\frac{\sum {\left(y_{i}^{exp}-y_{i}^{cal}\right)}^{2}}{n-k}\right)}^{1/2}$$

where *k* is the number of parameters fit by regression and n-*k* is the number of degrees of freedom of the regression.

The statistical study showed that for all the properties measured at 298.15 K, there were significant differences between the different systems with *p* < 0.0001 values.

From the chemical industry point of view, density is a very important basic feature for the evaluation not only of containers, the diameter of columns in industrial processes, etc., but also because density gives information about the purity of the chemicals, the interactions that are formed between the molecules that form the chemical system and the molecular structure in terms of the volume occupied by the molecules and free volume [31]. This parameter is essential in the extraction processes because it affects the kinetic rate and the driving forces between the solvents and the solid particles [35], or, in pharmaceutical formulations, provides critical information about the physical properties of drug substances and their formulations. This information can be used to optimize drug dosage, ensure physical stability and detect the presence of counterfeit or substandard drugs [36].

As it is well known, density values decrease as the temperature increases since volume increases with temperature. In this study, the highest values of density are found for FruGW followed by SorGW and XylGW. Concretely, at *T* = 298.15 K, density values are 1275.22 kg·m^−3^ for XylG, 1329.61 kg·m^−3^ for FruGW and 1300.25 kg·m^−3^ for SorGW. Density is affected by several factors such as the nature of the components forming the DES or composition [37]. The molecular organization and interactions between components of the mixture also play an important role in this property. Singh et al. prepared several DESs formed by choline chloride (ChCl) and polyethylene glycol 600 (PEG600) with glycerol at different molar ratios. The results then indicated that the density values of the studied DESs decrease with the presence of HBAs with respect to glycerol which presents a density value of 1253 kg·m^3^ at *T* = 298.15 K. This trend is the opposite of what we observed in our study, with the density of the DESs being higher than pure glycerol [38]. It is also noticeable that the density of the DESs containing sugars is higher than glyceline, a DES formed by ChCl and glycerol [39]. However, the values for this property are in the range of 1–1.3 kg·m^3^ according to literature data [40].

The molar volumes at *T* = 298.15 K for the studied DESs are 51.021, 52.445 and 53.881 cm^3^·mol^−1^ for XylGW, FruGW and SorGW, respectively. The molar volume values at low temperatures are lower since the average size of the holes created among the molecules is smaller at low temperatures, and therefore the density increases. 

One of the key properties required for the use of fluids in engineering applications is the isobaric expansivity α*_p_* which determines the temperature change in the volume V at constant pressure *p*. Thermal expansion is related to the asymmetry of the interatomic interactions so that the repulsive force grows faster than the attractive force. Upon transition to the liquid state, the bulk thermal expansion increases sharply [41].

The calculated isobaric expansivities, *α_p_*, for the studied DESs are 0.514, 0.537 and 0.498 kK^−1^ at *T* = 298.15 K for XylGW, FruGW and SorGW, respectively. These values are in the same range as other DESs published by Jafari et al. where they studied DESs formed by ChCl and ethylene glycol (ChCl:EG) at 1:2 and 1:5 molar ratios and mixtures with water (ChCl:EG:W) at 1:2:2 and 1:5:2. In this study, the values of this property were 0.499 for ChCl:EG (1:2) and 0.540 for ChCl:EG (1:5); when water is introduced into the system, the value of, *α_p_*, remains constant for (ChCl:EG:W) at 1:2:2 but increases in the case of (ChCl:EG:W) at 1:5:2 being 0.543 [42]. In the case of the binary system formed by choline chloride and glycerol (1:1.99), the value was 0.466, and with the inclusion of water, it was (1: 1.99:1.02) 0.465 kK^−1^ [39]. In the case of the work presented by Leron et al., this property is shown at 298.15 K for glyceline, and its value is 0.497 kK^−1^ [43].

The refractive index is a volumetric property that provides complementary information to density. For instance, one of the main uses of refractive index measurements in pharmaceutical formulation is to determine the purity and concentration of drug substances [44]. In addition, refractive index measurements can be used to monitor the physical stability of pharmaceutical formulations [45]. Changes in refractive index can indicate the formation of crystals or other solid phases that may affect the efficacy and safety of the drug.

As expected, when the temperature increases, refractive index values decrease and molar refraction values increase, both properties through a linear dependence. For the studied DESs, the highest values for the refractive index are found for FruGW followed by SorGW and XylGW, whereas for molar refraction, the trend is the following: SorGW > FruGW > XylGW. The property molar refraction is usually related to the hardcore volume of the thermodynamic system bulk. Thus, the free molar volume, i.e., the unoccupied volume, can also be calculated from the molar refraction and molar volume. In this case, the molar free volumes at T = 298.15 K are 36.887, 37.587 and 38.713 cm^3^·mol^−1^ for XylGW, FruGW and SorGW, respectively.

Another important physicochemical property for many industrial processes is surface tension, σ. It is a measure of the energy required to increase the surface area of a liquid by a given amount (the force that holds the molecules of a liquid together and prevents them from separating or spreading apart); this property therefore reflects the molecular attraction and interaction in the system [46]. An important aspect for many industrial processes is surface tension, σ. It is used to calculate the fluid flow for design equipment in mixing and separation processes [31]. This property depends on the intermolecular forces acting in the bulk phase compared to those presented on the surface. The surface tension decreases as the temperature increases because of the diminution in these bulk forces. That is, the higher the surface tension, the stronger the intermolecular interactions. For the studied DESs, the highest values of surface tension are found for SorGW, followed by FruGW and XylGW. The values obtained for this property are within the ranges found in the literature, which usually fluctuate between 35 and 75 mN·m^−1^ [40]. Furthermore, it has been shown in the literature that the surface tension values for sugar-based DESs, such as those formed by choline chloride and fructose [24] or choline chloride and glucose [47], are high due to the intermolecular interactions involved.

The studied volumetric properties also reflect the observed intensity of the intermolecular interactions, with a higher free volume when interactions are smaller (XylGW). The entropy, Δ*S_s_*, and enthalpy, Δ*H_s_*, of surface formation per unit surface area give information about the molecular distribution at the surface. In this case, the linear relationship between temperature and surface tension makes Δ*S*_s_ constant, the calculated values being equal to 0.2159, 0.2436 and 0.3916 mN·m^−1^·K^−1^ for XylGW, FruGW, and SorGW, respectively. At *T* = 298.15 K, the calculated Δ*H*_s_ values are 120.61, 129.86 and 178.61 mN·m^−1^ for XylGW, FruGW and SorGW, respectively. The orientation degree in the surface is indicated by low values of enthalpy and entropy of surface formation. Thus, XylGW seems to be the moiety with a higher surface orientation degree.

In Figure 2, the speed of sound and isentropic compressibility as a function of temperature are shown. The speed of sound is a quite useful property used in some applications such as the development of equations of state constants, bulk modulus, heat capacity or Joule–Thomson coefficient, among others [48]. The speed of sound is related to the efficiency of molecular packing. Substances that present higher speed of sound values are packed more effectively at a molecular level. In this case, the observed behavior of the speed of sound follows the typical inverse relation with temperature, and experimental values decrease when the temperature increases. Clearly, the speed of sound values for XylGW are lower than those for FruGW and SorGW. 

Some important information about the internal organization of the eutectic mixture can be obtained through isentropic compressibility. High values of this property indicate that the chemical structure is less packed. In this case, the isentropic compressibility values increase as the temperature does. For the studied DESs, the highest values are found for XylGW followed by SorGW and FruGW.

The isobaric molar heat capacity, *C*_p,m_, is directly related to the heat transfer. This property increases as the temperature does. Higher values are found for SorGW followed by FruGW and XylGW.

Finally, the fluidity of the studied DESs has been obtained through kinematic viscosity, *ν*. Using experimental density values, the dynamic viscosity property, *η*, was obtained. Viscosity is a transport property that describes the internal resistance of a fluid to shear stress [49]. It is a mole- and temperature-dependent property. It is important to establish a viscosity database as this property is directly related to fluid flow calculations and equipment design. With these data at the design stage, the DES compound can be designed to achieve lower viscosity values [50].

Most of the deep eutectic solvents reported so far are highly viscous at room temperature (*η* > 100 mPa·s), which is mainly due to the extensive hydrogen bonding network between the components of the deep components of eutectic solvents. They also exhibit a wide viscosity range [40]. In the case of the studied DESs, the dynamic viscosity presents values in the range of 100–4000 mPa·s at the studied temperatures. The dependency of viscosity with the temperature, water content, nature of its components and molar ratio in the DESs has been previously studied [40]. In this case, the highest values for dynamic viscosity are found for FruGW followed by SorGW and XylGW. These viscosity values are quite high compared to other DES-containing glycerols such as glyceline (ChCl + glycerol 1:2) or ChCl + PEG600 + glycerol (1:3:2, 1:4:2 and 1:5:2) with dynamic viscosity values between 100 and 700 mPa·s [38,39] at the same temperature range as the measurements of the present work. This is significant since the DESs studied in this work contain water, which translates into a reduction in viscosity.

## 3. Materials and Methods

### 3.1. Chemicals

**Figure 4:**
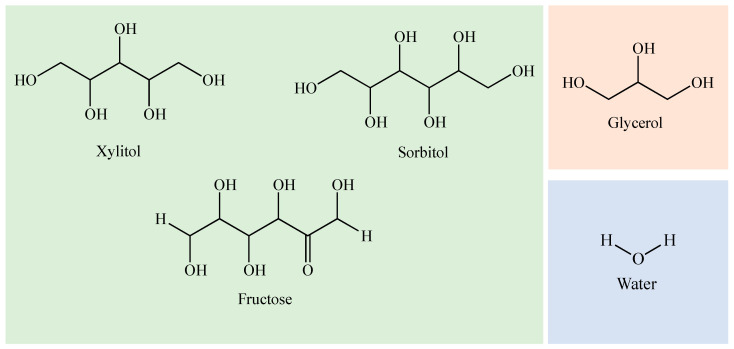
Chemical structures of the components of DESs.

Some information on the pure components used in this work is gathered in Table 2. Chemical structures are presented in Figure 4. The chemicals were dried under vacuum for 24 h prior to use. 

These components were chosen because they are common compounds in the pharmaceutical industry and it may be of interest to know how these systems behave so that they can be used in the future as drug delivery vehicles, for example via the transdermal or rectal routes.

### 3.2. Preparation of Deep Eutectic Solvents

In this study, 50 mL of three DESs were prepared. Briefly, components for each system were weighed using a Sartorius Entris 5201-1S balance (uncertainty ± 10^−1^ g) and introduced into a jar with constant stirring and heating in a water bath at 60–70 °C until a mixture of transparent and homogeneous appearance was obtained [25]. In order to avoid water uptake and to ensure the ratio, the properties were measured as soon as the compounds were prepared.

Table 3 shows all the initially prepared systems. It can be seen that all the analyzed systems were liquid at room temperature and 4 °C, so a decrease in melting temperature was observed. The mixtures were very viscous and difficult to work with; it was decided to add water to make them easier to handle. Of all the ternary systems containing water, those marked with an asterisk* were selected to be studied in depth because they were less viscous than the others. Therefore, DESs formed via the combination of xylitol, fructose and sorbitol with glycerol and water in the molar ratio 1:2:3 have been studied. Once the compounds were prepared, the properties were measured.

Information related to the composition (molar ratio) and the final molar mass of the mixture can be found in Table 4. The molar mass of each mixture was calculated using the following equation:

MW_DES_ = X_glycerol_·MW_glycerol_ + X_Sugar_ ·MW_Sugar_ + X_water_·MW_water._ (4)

where *X* is the mole fraction and *MW* is the molar mass.

### 3.3. Thermophysical Properties

Some physicochemical properties such as the refractive index, the speed of sound, the density, the viscosity, the surface tension and the isobaric molar heat capacity were obtained. All the values were measured in 2.5 K intervals in the range of temperatures (278.15–338.15). Several derived properties (isentropic compressibility, enthalpy of surface formation and entropy of surface formation and isobaric expansivity) were also calculated from the raw experimental data. All properties were measured in triplicate (n = 3).

An Anton Paar DSA 5000 vibrating tube sound analyzer and densimeter (3 MHz) was used to simultaneously measure the density, *ρ*, and speed of sound, *u*, of the studied DESs. Dry air and ultrapure water (SH Calibration Service GmbH) were used for calibrating the equipment. The temperature was controlled internally, with an uncertainty of 0.005 K. The uncertainty of the measurements is the following: for the speed of sound, 0.5 m·s^−1^, and for density measurements, 0.1 kg·m^−3^. The isobaric expansivity, *α_p_*, was calculated as follows: $\alpha_{P}=-\left(1/\rho \right){\left(d\rho /dT\right)}_{P}$, while the isentropic compressibility, κ*_s_*, can be calculated as $\kappa_{s}=1/(\rho u^{2})$, assuming that the ultrasonic absorption is zero.

The Abbemat-HP refractometer Dr Kernchen was used for measuring the refractive indices, *n*_D_ (sodium D wavelength 589.3 nm) whose temperature was internally controlled (±0.01 K), the uncertainty in the measurement being 5·10^−5^. The molar refraction, *R_m_*, was also calculated: $R_{m}=M(n_{D}^{2}-1)/\left(\rho \left(n_{D}^{2}+1\right)\right)$.

A Lauda TVT-2 drop volume tensiometer was used to measure the surface tensions, *σ*, of the studied DESs. The temperature was controlled with a Lauda E-200 thermostat (±0.01 K). The uncertainty of the measurement in this case is 0.2 mN·m^−1^. In order to obtain information about the molecular distribution in the surface, both the entropy, *ΔS_s_*, and enthalpy, *ΔH_s_*, of the surface formation per unit surface area were calculated using ${\Delta S}_{s}=-{\left(d\sigma /dT\right)}_{P}$ and ${\Delta H}_{s}=\sigma -T{\left(d\sigma /dT\right)}_{P}$, respectively.

TA Instruments Q2000 differential scanning equipment was used to obtain experimental isobaric molar heat capacities, *C*_p,m_, with an uncertainty of 1%.

The kinematic viscosities, *ν*, were measured with Schoot-GeräteAVS-440 equipment and Ubbelohde capillary viscosimeters, applying kinetic corrections. The uncertainty for this property is 0.01 s, and the temperature was controlled using a thermostat model Schoot-Geräte CT 1150/2 ± 0.01 K. The dynamic viscosity, *η*, was calculated from experimental kinematic viscosity data, η=ρν, with 1% uncertainty.

### 3.4. Statistical Analysis

The statistical analysis was conducted utilizing GraphPad Prism 9.0 software, employing the one-way ANOVA method and the Tukey-Kramer honestly significant differences model. The null hypothesis (H0) posits that there are no significant differences among the groups, and, therefore, they are equal. Conversely, the alternative hypothesis (H1) assumes that there are differences between the groups. A confidence level of 95% was chosen, implying that if the *p*-value was less than 0.05, the null hypothesis was rejected, and the alternative hypothesis was accepted.

## 4. Conclusions

In this paper, the preparation and complete thermophysical characterization of three DESs formed by sugar (xylitol, fructose and sorbitol), glycerol and water in the molar ratio of 1:2:3 were carried out. The experimental values of density, refractive index, the speed of sound, surface tension, isobaric molar heat capacity and kinematic viscosities were obtained in the range of temperatures (278.15–338.15) K at atmospheric pressure. Additionally, the derived properties such as molar refraction, isentropic compressibility and dynamic viscosities were calculated. For these DESs, a linear dependence between property and temperature was obtained for density, refractive index, molar refraction, surface tension and isobaric heat capacity. However, this relation was polynomial for the case of the speed of sound and temperature. For the eutectic systems studied, density data ranging from 1312.62–1248.78 kg·m^−3^ were obtained, depending on the temperature analyzed, and the system that presented the highest values was FruGW. In the case of isobaric expansivity, α_p_, the values varied between 0.498 and 0.537 kK^−1^ at 298.15 K. These values are similar to those obtained by other DESs containing glycerol in their systems.

The range of refractive index values found for these systems varies between 1.456012 and 1.482363, with FruGW being the one with the highest values. Surface tension, which is a very important property in industrial processes, is directly related to the intermolecular forces of mixtures [51]. In this case, these forces are much stronger for the system formed by sorbitol (SorGW) and weaker for that formed by xylitol (XylGW). In addition to analyzing the internal organization of the DESs through isentropic compressibility, it was observed that the one with the highest values is XylGW, indicating that its chemical structure is more compact than the other two systems.

In terms of C_p,m_, it was observed that the DES with sorbitol (SorGW) provided the highest heat transfer.

Finally, in the case of dynamic viscosity, the values were fitted to a Vogel–Fulcher–Tammann equation. It is important to note that all have high viscosity values, with FruGW and SorGW being more viscous than XylGW.

Deep eutectic solvents with higher density and refractive index values such as those of this study containing sorbitol could be used for the extraction of heavy metals [52]. With regard to the processing of lignocellulosic mass, it is generally known that a better penetration into the delignified raw material is achieved if the used solvent shows a lower density and viscosity [53]. Viscosity is also an important property related to some industrial applications of DESs [47].

On the other hand, solvents with lower values of density and refractive index, such as those containing xylitol, could be more easily used for the development of liquid or semisolid pharmaceutical and cosmetic forms [54]. This type of solvent could also be used in the food industry as texture agents and stabilizers [55].

With regard to industrial applications, isobaric molar heat capacity, *C_p_*_,*m*_, assumes importance (temperature control, optimizing reaction kinetics, energy efficiency, reaction rates, aids predictive modeling, drying and cooling processes and guides thermal energy storage systems). DESs with greater *C_p_*_,*m*_, such as sorbitol ones, could find utility in endothermic and chemical reaction processes thanks to their capacity to absorb and retain heat, thus enhancing their reaction efficiency and control [56]. Furthermore, these DESs could be harnessed for heat storage systems and refrigeration/climatization technologies, effectively absorbing and releasing heat, thereby contributing to efficient thermal energy conservation and controlled dissemination. On the other hand, DESs with lower *C_p_*_,*m*_ could optimize exothermic reactions, offer robust cooling in refrigeration, accelerate freezing in food production, and efficiently manage heat in thermal storage, including solar energy contexts [57].

In general, DESs with higher viscosity values can also be used as efficient lubricants in machinery, particularly under high-stress conditions [58]. On the contrary, if the viscosity is lower, their industrial applications could be solvent extraction and reaction solvents, facilitating better mixing and heat transfer in chemical reactions. In the field of coatings and inks, DESs with lower viscosities can provide improved spreading and coating uniformity [59].

## Figures and Tables

**Table 1:** Fitting parameters, *A*, *B* and *C*, for the correlation equations and their corresponding standard deviations, *S*_yx_.

Property	*A*	*B*	*C*	*S*_yx_
Xylitol: glycerol: water (1:2:3)				
*ρ*/(kg·m^−3^)	−0.6488	1468.51		0.182
*u*/(m·s^−1^)	−2.184	2612.57		0.291
*n*_D_	−0.000248	1.540010		10 × 10^−5^
*σ*/(mN·m^−1^)	−0.2159	120.47		0.115
*C*_p,m_/(J·mol^−1^·K^−1^)	0.303	64.4		0.097
*η ^a^*/(mPa·s)	0.00678	1424.4	164.87	4.884
Fructose: glycerol: water (1:2:3)				
*ρ*/(kg·m^−3^)	−0.7056	1539.80		0.251
*u*/(m·s^−1^)	−2.505	2733.67		0.481
*n*_D_	−0.000273	1.559727		13 × 10^−5^
*σ*/(mN·m^−1^)	−0.2436	130.01		0.187
*C*_p,m_/(J·mol^−1^·K^−1^)	0.462	21.9		0.126
*η ^a^*/(mPa·s)	0.00033	2332.3	139.53	5.980
Sorbitol: glycerol: water (1:2:3)				
*ρ*/(kg·m^−3^)	−0.6412	1491.30		0.187
*u*/(m·s^−1^)	−2.305	2681.22		0.334
*n*_D_	−0.000253	1.550538		5 × 10^−5^
*σ*/(mN·m^−1^)	−0.3916	174.78		0.181
*C*_p,m_/(J·mol^−1^·K^−1^)	0.323	72.1		0.130
*η ^a^*/(mPa·s)	0.00340	1692.9	160.20	8.076

*^a^ A* = *η*_0_; *C* = *T*_0_. Density, *ρ*, speed of sound, *u*,refractive index, *n*_D_, surface tension, *σ*, isobaric molar heat capacity, *C*_p,m_, dynamic viscosity, *η*.

**Table 2:** Information on the pure chemicals used in this study.

Chemical	CAS Number	Formula	Supplier	Purity (Mass Fraction)
Xylitol	87-99-0	C_5_H_12_O_5_	Fagron Iberica, Zaragoza, Spain	0.997
Fructose	57-48-7	C_6_H_12_O_6_	Sigma-Aldrich, Darmstadt, Germany	0.999
Sorbitol	50-70-4	C_6_H_14_O_6_	Sigma-Aldrich, Darmstadt, Germany	0.990
Glycerol	56-81-5	HOCH_2_CH(OH)CH_2_OH	Sigma-Aldrich, Darmstadt, Germany	0.999

**Table 3:** Composition, molar ratio, and physical appearance of the prepared DESs.

DES	Ratio	Physical Appearance
Xylitol:glycerol	1:2	Transparent, very viscous liquid
Fructose:glycerol	1:2	Transparent, very viscous liquid
Sorbitol:glycerol	1:2	Transparent, very viscous liquid
Xylitol:glycerol	1:3	Transparent, very viscous liquid
Fructose:glycerol	1:3	Transparent, very viscous liquid
Sorbitol:glycerol	1:3	Transparent, very viscous liquid
Xylitol:glycerol: water	1:3:1	Transparent, very viscous liquid
Xylitol:glycerol: water	1:3:2	Transparent, very viscous liquid
Xylitol:glycerol: water	1:3:3	Transparent, very viscous liquid
Fructose:glycerol:water	1:3:1	Transparent, very viscous liquid
Fructose:glycerol:water	1:3:2	Transparent, very viscous liquid
Fructose:glycerol:water	1:3:3	Transparent, very viscous liquid
Sorbitol:glycerol:water	1:3:1	Transparent, very viscous liquid
Sorbitol:glycerol:water	1:3:2	Transparent, very viscous liquid
Sorbitol:glycerol:water	1:3:3	Transparent, very viscous liquid
Xylitol:glycerol: water	1:2:1	Transparent, viscous liquid
Xylitol:glycerol: water	1:2:2	Transparent, viscous liquid
Xylitol:glycerol: water *	1:2:3	Transparent, slightly viscous liquid
Fructose:glycerol:water	1:2:1	Transparent, viscous liquid
Fructose:glycerol:water	1:2:2	Transparent, viscous liquid
Fructose:glycerol:water *	1:2:3	Transparent, slightly viscous liquid
Sorbitol:glycerol:water	1:2:1	Transparent, viscous liquid
Sorbitol:glycerol:water	1:2:2	Transparent, viscous liquid
Sorbitol:glycerol:water *	1:2:3	Transparent, slightly viscous liquid

**Table 4:** Deep eutectic systems: abbreviation, molar ratio and molar mass.

DES	Abbreviation	Molar Ratio	Molar Mass (g·mol^−1^)
Xylitol: glycerol: water	XylGW	1:2:3	65.063
Fructose: glycerol: water	FruGW	1:2:3	69.732
Sorbitol: glycerol: water	SorGW	1:2:3	70.068

## Data Availability

On request.

## Supplementary Materials

The following supporting information can be downloaded at: https://www.mdpi.com/article/10.3390/molecules28166023/s1, Table S1. Experimental and some derived thermophysical properties of deep eutectic solvents as a function of the temperature, *T*, at atmospheric pressure, *p* = 0.1 MPa.

## Sample Availability

Not available.
