# Supplementary material for: Deep Eutectic Solvents Formed by Glycerol and Xylitol, Fructose and Sorbitol: Effect of the Different Sugars in Their Physicochemical Properties
Source: Molecules. 2023 Aug 11;28(16):6023. doi: 10.3390/molecules28166023 (PMC10458529; doi:10.3390/molecules28166023)
Supplement: Supplementary file 1 [file molecules-28-06023-s001.zip › molecules-2532631-supplementary.pdf]

Table S1. Experimental and some derived thermophysical properties of deep eutectic solvents as a function of the temperature, T, at atmospheric pressure, p = 0.1 MPa.[a]

T / K	ρ / (kg·m^{-3})	u / (m·s^{-1})	κ_S / TPa^{-1}	n_D	R_m / (cm^3·mol^{-1})	σ / (mN·m^{-1})	$C_{p.m}$ / (J·mol-1·K^{-1})	ν / (mm^2·s^{-1})	η / (mPa·s)
				Xylitol: glycerol: water (1:2:3)					
278.15	1287.71					60.54	148.8	1525	1964
280.65	1286.17					59.90	149.6	1160	1492
283.15	1284.62			1.469597	14.120	59.23	150.1	882.6	1134
285.65	1283.06			1.469056	14.124	58.82	151.0	700.1	898.2
288.15	1281.50			1.468507	14.127	58.35	151.7	556.0	712.5
290.65	1279.92			1.467923	14.129	57.64	152.5	443.0	567.0
293.15	1278.36			1.467329	14.131	57.32	153.2	355.5	454.5
295.65	1276.80			1.466738	14.133	56.64	153.9	287.7	367.3
298.15	1275.22	1961.35	203.85	1.466138	14.134	56.24	154.8	232.7	296.7
300.65	1273.63	1955.89	205.24	1.465541	14.137	55.51	155.5	191.1	243.4
303.15	1272.03	1950.39	206.66	1.464932	14.138	55.07	156.4	159.1	202.4
305.65	1270.41	1945.00	208.07	1.464318	14.140	54.47	157.0	132.7	168.5
308.15	1268.79	1939.63	209.49	1.463704	14.142	53.93	157.8	111.8	141.9
310.65	1267.16	1934.27	210.93	1.463084	14.144	53.27	158.6	93.94	119.0
313.15	1265.51	1928.90	212.38	1.462470	14.146	52.80	159.4	80.25	101.6
315.65	1263.87	1923.52	213.85	1.461846	14.148	52.24	160.2	68.19	86.18
318.15	1262.22	1918.13	215.33	1.461212	14.150	51.70	160.7	58.83	74.25
320.65	1260.56	1912.71	216.84	1.460577	14.152	51.12	161.7	43.23	54.50
323.15	1258.90	1907.25	218.37	1.459927	14.153	50.60	162.4	40.06	50.43
325.65	1257.23	1901.75	219.93	1.459285	14.155	49.97	163.1	35.33	44.42
328.15	1255.56	1896.21	221.51	1.458636	14.157	49.50	163.8	31.17	39.13

Table S1. Continuation.

T / K	ρ / (kg·m^{-3})	u / (m·s^{-1})	κ_S / TPa^{-1}	n_D	R_m / (cm^3·mol^{-1})	σ / (mN·m^{-1})	$C_{p.m}$ / (J·mol-1·K^{-1})	ν / (mm^2·s^{-1})	η / (mPa·s)
330.65	1253.87	1890.62	223.12	1.457979	14.158	49.26	164.6	27.67	34.69
333.15	1252.18	1884.99	224.76	1.457326	14.160	48.71	165.4	24.68	30.91
335.65	1250.49	1879.34	226.42	1.456674	14.162	48.16	166.3	22.02	27.54
338.15	1248.78	1873.63	228.11	1.456012	14.163	47.61	166.8	19.75	24.66
Fructose: glycerol: water (1:2:3)									
278.15	1343.18					62.40	150.6	4986	6697
280.65	1341.46					61.76	151.5	3707	4973
283.15	1339.69			1.482363	14.849	61.21	152.8	2785	3731
285.65	1337.90			1.481670	14.850	60.54	153.8	2116	2831
288.15	1336.36			1.481095	14.852	60.05	154.9	1619	2163
290.65	1334.80			1.480486	14.854	59.13	156.1	1247	1664
293.15	1333.22			1.479862	14.855	58.67	157.2	963.8	1285
295.65	1331.53			1.479215	14.856	57.79	158.5	765.3	1019
298.15	1329.61	1987.57	190.38	1.478453	14.858	57.23	159.9	604.7	804.2
300.65	1327.92	1981.06	191.88	1.477790	14.859	56.74	160.7	481.7	639.9
303.15	1326.14	1974.90	193.34	1.477130	14.861	55.90	161.9	378.8	502.4
305.65	1324.42	1968.24	194.90	1.476440	14.862	55.49	163.2	305.9	405.2
308.15	1322.62	1961.81	196.45	1.475784	14.865	54.95	164.5	249.7	330.3
310.65	1320.86	1955.01	198.08	1.475079	14.866	54.25	165.3	205.6	271.5
313.15	1319.05	1948.60	199.66	1.474413	14.868	53.42	166.5	170.6	225.0
315.65	1317.34	1942.39	201.20	1.473740	14.869	53.17	167.7	142.6	187.9
318.15	1315.51	1936.24	202.76	1.473044	14.871	52.40	168.9	120.0	157.9
320.65	1313.67	1930.10	204.34	1.472352	14.873	51.99	170.2	101.6	133.5
323.15	1311.82	1924.00	205.93	1.471641	14.875	51.02	171.1	86.64	113.7

Table S1. Continuation.

T / K	ρ / (kg·m^{-3})	u / (m·s^{-1})	κ_S / TPa^{-1}	n_D	R_m / (cm^3·mol^{-1})	σ / (mN·m^{-1})	$C_{p.m}$ / (J·mol-1·K^{-1})	ν / (mm^2·s^{-1})	η / (mPa·s)
325.65	1309.96	1917.89	207.54	1.470917	14.877	50.51	172.3	74.18	97.17
328.15	1308.11	1911.80	209.16	1.470218	14.879	49.86	173.5	64.02	83.74
330.65	1306.25	1905.69	210.80	1.469483	14.880	49.74	174.9	55.50	72.50
333.15	1304.48	1899.54	212.45	1.468796	14.881	49.11	175.8	48.48	63.24
335.65	1302.71	1893.36	214.13	1.468130	14.884	48.49	177.0	42.42	55.25
338.15	1300.98	1887.18	215.83	1.467493	14.886	47.86	178.0	37.35	48.58
				Sorbitol: glycerol: water (1:2:3)					
278.15	1312.62					69.97	161.9	4435	5821
280.65	1311.10					68.99	162.6	3277	4296
283.15	1309.57			1.478796	15.167	67.93	163.4	2485	3254
285.65	1308.04			1.478168	15.168	66.93	164.3	1891	2474
288.15	1306.49			1.477599	15.170	65.87	165.2	1457	1904
290.65	1304.95			1.476977	15.171	64.92	166.1	1128	1472
293.15	1303.39			1.476333	15.172	63.93	166.8	882.3	1150
295.65	1301.82			1.475696	15.173	62.95	167.5	695.2	905.0
298.15	1300.25	1994.45	193.34	1.475098	15.175	61.85	168.2	553.0	719.1
300.65	1298.66	1988.48	194.74	1.474470	15.176	60.89	169.2	442.5	574.7
303.15	1297.09	1982.34	196.19	1.473853	15.177	59.90	169.8	358.6	465.1
305.65	1295.51	1976.29	197.63	1.473231	15.179	59.16	170.8	292.9	379.4
308.15	1293.93	1970.43	199.05	1.472610	15.180	58.13	171.5	241.1	312.0
310.65	1292.34	1964.64	200.47	1.471971	15.181	57.35	172.5	199.8	258.2
313.15	1290.73	1958.92	201.90	1.471345	15.183	55.90	173.2	167.3	216.0
315.65	1289.10	1953.23	203.33	1.470698	15.184	55.10	173.8	141.3	182.2
318.15	1287.47	1947.54	204.78	1.470057	15.186	53.91	174.6	118.4	152.4

Table S1. Continuation.

T / K	ρ / (kg·m^{-3})	u / (m·s^{-1})	κ_S / TPa^{-1}	n_D	R_m / (cm^3·mol^{-1})	σ / (mN·m^{-1})	$C_{p.m}$ / (J·mol-1·K^{-1})	ν / (mm^2·s^{-1})	η / (mPa·s)
320.65	1285.82	1941.86	206.25	1.469422	15.188	52.94	175.6	107.2	137.9
323.15	1284.17	1936.17	207.73	1.468768	15.189	51.97	176.5	92.65	119.0
325.65	1282.51	1930.48	209.22	1.468138	15.191	50.98	177.1	80.11	102.7
328.15	1280.85	1924.77	210.74	1.467479	15.192	50.02	178.1	69.27	88.72
330.65	1279.18	1919.04	212.28	1.466839	15.194	49.54	178.8	60.31	77.15
333.15	1277.50	1913.29	213.83	1.466187	15.196	48.54	179.6	52.64	67.24
335.65	1275.81	1907.50	215.42	1.465526	15.198	47.54	180.5	46.27	59.04
338.15	1274.12	1901.67	217.03	1.464875	15.199	46.54	181.3	40.92	52.13

[a] Standard uncertainties u are $u(T)$ = 0.005 K for density and speed of sound and $u(T)$ = 0.01 K for the rest of properties, $u(p)$ = 1 kPa, and the combined expanded uncertainties U_c are $U_c(\rho)$ = 0.1 kg·m^{-3}, $U_c(u)$ = 0.5 m·s^{-1}, $U_c(n_D)$ = 5·10^{-5}, $U_c(\sigma)$ = 0.5 mN·m^{-1}, $U_c(\nu)$ = 1 %, $U_c(\eta)$ = 1 %, $U_c(\kappa)$ = 1 %, with 0.95 level of confidence (k = 2).
